# Always available? A systematic review on extended work-related availability, health outcomes and work-family conflict

**DOI:** 10.3389/fpsyg.2025.1726421

**Published:** 2026-01-12

**Authors:** Simon Renk, Christine Sutter

**Affiliations:** Institute of Traffic and Engineering Psychology, German Police University, Münster, Germany

**Keywords:** job demands-resources model, work-life border theory, conservation of resources theory, work-related information and communication technology use, well-being

## Abstract

**Introduction:**

This study critically explores the link between work-related extended availability, work-family conflict, and health outcomes through a systematic literature review, incorporating various theoretical frameworks, including the Work-Life Border Theory, the Job Demands-Resources Model, and the Conservation of Resources Theory.

**Methods:**

Following PRISMA 2020 guidelines, 17 studies were selected for the final analysis.

**Results:**

The review shows that higher levels of work-related extended availability are associated with increased work-family conflict and poorer health outcomes, notably higher stress, burnout, and reduced mental well-being. The research identifies key moderating factors such as organizational conditions (like structural autonomy and workload control) and boundary management style, which can lessen or intensify the negative impacts of work-related extended availability.

**Discussion:**

These results deepen the understanding of the complex, conditional relationship between work-related extended availability and work-family conflict, highlighting the need for nuanced, context-aware strategies that address individual differences and organizational settings. This work provides valuable theoretical insights and practical guidance for managing the challenges of work-related extended availability in modern organizations. For future studies, examining additional moderation effects is recommended, employing longitudinal designs, and broadening the research scope to include high-risk professions.

## 1 Introduction

### 1.1 Theoretical and practical context of work-related extended availability

Technological advancements have enabled employees to remain virtually connected to work and to be available outside traditional office hours. Work-related extended availability refers to the expectation or practice of employees being accessible and responsive to work-related matters outside of regular working hours, for instance, by work-related information and communication technology use during personal time. Work-related extended availability blurs the line between work and personal life, and, as a consequence, might trigger positive or negative outcomes for employees and organizations. Research shows that work-related extended availability can offer advantages such as increased flexibility and responsiveness to global collaboration requirements, enabling employees to manage tasks across time zones and adapt to changing working conditions (Dettmers et al., 2016). However, these benefits can also have drawbacks: increased availability often promotes the expectation of constant connectivity, which in turn is associated with negative effects on employee well-being (Dettmers et al., 2016; Ziebertz et al., 2015). For instance, prior research suggests that continual connectivity and work-related use of information and communication technology may contribute to increased emotional exhaustion (Tedone, 2022), overall deterioration in health outcomes (Pluut and Wonders, 2020), and work-family conflict (Sousa et al., 2023). Work-family conflict refers to a form of role conflict in which the demands of work and family roles are incompatible, making it difficult to perform one role while simultaneously fulfilling the other (Greenhaus and Beutell, 1985). Furthermore, the perception of technology as a demand, which often accompanies after-hours communication, may contribute to higher levels of burnout and turnover among employees (Rasulova and Tanova, 2025). Despite growing empirical evidence (Korunovska and Spiekermann-Hoff, 2019; Thörel et al., 2022), the exact psychological and organizational mechanisms through which work-related extended availability impairs employee well-being, especially by negative consequences such as emotional exhaustion, sleep disorders, and conflicts between work and family, remain controversial. In particular, it is unclear under which conditions these adverse effects can be mitigated or even reversed, for example, through individual dispositions or organizational circumstances.

From a methodological point of view, work-related extended availability is often measured using a a quantitative approach. Many researchers (e.g., Pauls et al., 2017) typically draw on self-report surveys measuring the frequency and amount of communication and work-related activities carried out outside the employees’ normal work schedule: “*How many hours (on average) during your leisure time did you spend assisting with any work-related phone calls, emails, or messages?*.” Operationalizing work-related extended availability can also include evaluating structured on-call shifts where the employee is expected to be available to respond to work-related issues after business hours, including nights and weekends (Dettmers et al., 2016). The researchers would assess the extended availability separately for weeks worked during the participants’ on-call responsibilities and for other weeks and time off when they were not on call. With a more qualitative approach (e.g., Dettmers et al., 2016), employees are explicitly asked about how much they feel formally or informally expected to be available for work outside regular hours. The rapidly growing body of research concerning the effects of work-related extended availability on employees relies almost exclusively on samples from generic office settings, education, or mixed-industry panels. The consensus across studies is high: engaging with work-related information and communication technology outside formal hours consistently undermines recovery and harms employee well-being. Large cross-sectional evidence already links pervasive after-hours connectivity to higher fatigue and emotional exhaustion (Dettmers et al., 2016; Ziebertz et al., 2015). Daily diary studies extend this pattern, showing that when employees use smartphones or email for work in the evenings, they experience poorer sleep and report greater next-day burnout and reduced well-being (Gombert et al., 2017). Experimental data provide a cognitive explanation: simply perceiving an urgent need to respond to late-night messages increases stress and lowers subjective well-being, even before any actual task is completed (Giurge and Bohns, 2021). Meta-analyses and systematic reviews generally agree on moderate positive associations between after-hours information and communication technology use and both emotional exhaustion and sleep problems (Blake et al., 2024; Baumeister et al., 2021). At the same time, the effects on task performance are neutral to slightly negative once exhaustion is accounted for (Chen and Karahanna, 2018). Overall, these findings portray work-related extended availability as a persistent boundary-crossing demand that hinders recovery, harms mental health, and does not consistently improve performance, contrary to common managerial belief. High-reliability organizations (HROs), such as the military, police forces, aviation control centers, and emergency medicine units, have rarely been studied in this context (e.g., Scholarios et al., 2018; Vila, 2006). Yet, these environments are where the impacts of after-hours connectivity are most severe. According to Scholarios et al. (2018), for the police service, demands for operational efficiency, shift work, and additional working hours are an expectation. However, extended, unpredictable shifts are seldom officially recognized. The research by Vila (2006) confirms that police officers often are overly fatigued because of long and erratic work hours, shift work, and insufficient sleep. Moreover, shift work and long work hours across a career in law enforcement negatively impact sleep and increase the likelihood of on-duty fatigue and performance impairment (Riedy et al., 2021). From the Job Demands-Resources (JD-R) Model (Bakker et al., 2023), we would predict that work demands in HROs (e.g., constant availability, extended working hours, and the resulting potential increase in operational risk and stress) aggravate the aforementioned effects of work-related extended availability on police and military personnel. Studying work-related extended availability in these settings would, therefore, illuminate boundary-management processes under extreme accountability, examine whether the established links to exhaustion and impaired recovery generalize, and guide evidence-based policy on digital curfews and right-to-disconnect provisions for critical personnel’s performance.

The current review aims to synthesize existing knowledge on work-related extended availability and its impacts on employee well-being in a generic work context. The review’s focus will be set on the influence of moderating and mediating effects to deepen the understanding of the relationship between work-related extended availability and health outcomes. The theoretical perspectives, such as the JD-R Model (Demerouti et al., 2001; Schaufeli and Taris, 2014), Work-Life Border Theory (Clark, 2000), and Conservation of Resources Theory (Buchwald and Hobfoll, 2004), offer complementary insights into how work-life boundaries are managed, how job stressors lead to resource depletion, and how these dynamics ultimately result in adverse health outcomes.

### 1.2 Work-leisure as the overarching context

The theoretical and conceptual research on work-related extended availability is grounded in several key frameworks. For understanding the interplay between job characteristics and employee well-being and motivation on organizational outcomes, the JD-R model has become one of the most influential and well validated models in organizational psychology (Demerouti et al., 2001; Schaufeli and Taris, 2014). The model assumes that all psychosocial work characteristics can be classified into demands and resources (Bakker and Demerouti, 2007). Job demands relate to work’s psychological, physical, and social aspects, which require certain skills or efforts and are associated with certain costs (Demerouti and Nachreiner, 2019). Job resources encompass physical, psychosocial, and organizational elements of work that facilitate goal achievement and encourage personal growth, learning, and development (Demerouti et al., 2001). Whether a psychosocial work characteristic (e.g., social support from a colleague) is experienced as a demand (e.g., intrusive) or resource (e.g., supportive or empathic) varies individually and changes depending on contextual factors. Social support as an experienced job resource is expected to help in dealing with inadequate work-recovery time and the impact of job-related stressors. A widely held notion known as the “buffering hypothesis” (Cohen and Wills, 1985) suggests that social support as a resource can mitigate the potentially harmful effects of job stressors on employee health, well-being, and work engagement.

The Work-Life Border Theory, initially proposed by Clark (2000), broadens the theoretical perspective and understands individuals as “border-crossers” who navigate between work and personal domains. The theory emphasizes the differences in purpose, culture, and expected behaviors between these domains, necessitating active boundary management. Key features of these borders include flexibility and permeability, which refer to the ability to expand boundaries and the degree to which one domain interferes with another. Successful transitions between domains rely on establishing clear markers or cues that distinguish one domain from another (Clark, 2000). Subsequent research has expanded this framework. Nippert-Eng and Voydanoff (1998) provided foundational work on boundary negotiation, highlighting how individuals construct and maintain boundaries through everyday practices. Kreiner et al. (2009) identified specific boundary work tactics, while Rothbard et al. (2005) emphasized the importance of individual preferences in boundary management. These preferences, shaped by organizational cultures, job demands, and family responsibilities, significantly impact work satisfaction and well-being (Kossek and Lautsch, 2012). The Conservation of Resources Theory, proposed by Hobfoll (1989), complements boundary theories by emphasizing that individuals seek to acquire, maintain, and safeguard their valued resources. According to the Conservation of Resources Theory, stress occurs when there is either a threat of resource loss, actual loss, or insufficient resource gain following significant investment. Work-related extended availability may contribute to the depletion of physical and intangible resources, leading to a “loss spiral” that results in burnout and stress (Buchwald and Hobfoll, 2004). Empirical research supports this idea, indicating that the inability to replenish resources is a key driver of stress (Heath et al., 2012).

**Figure 1:**
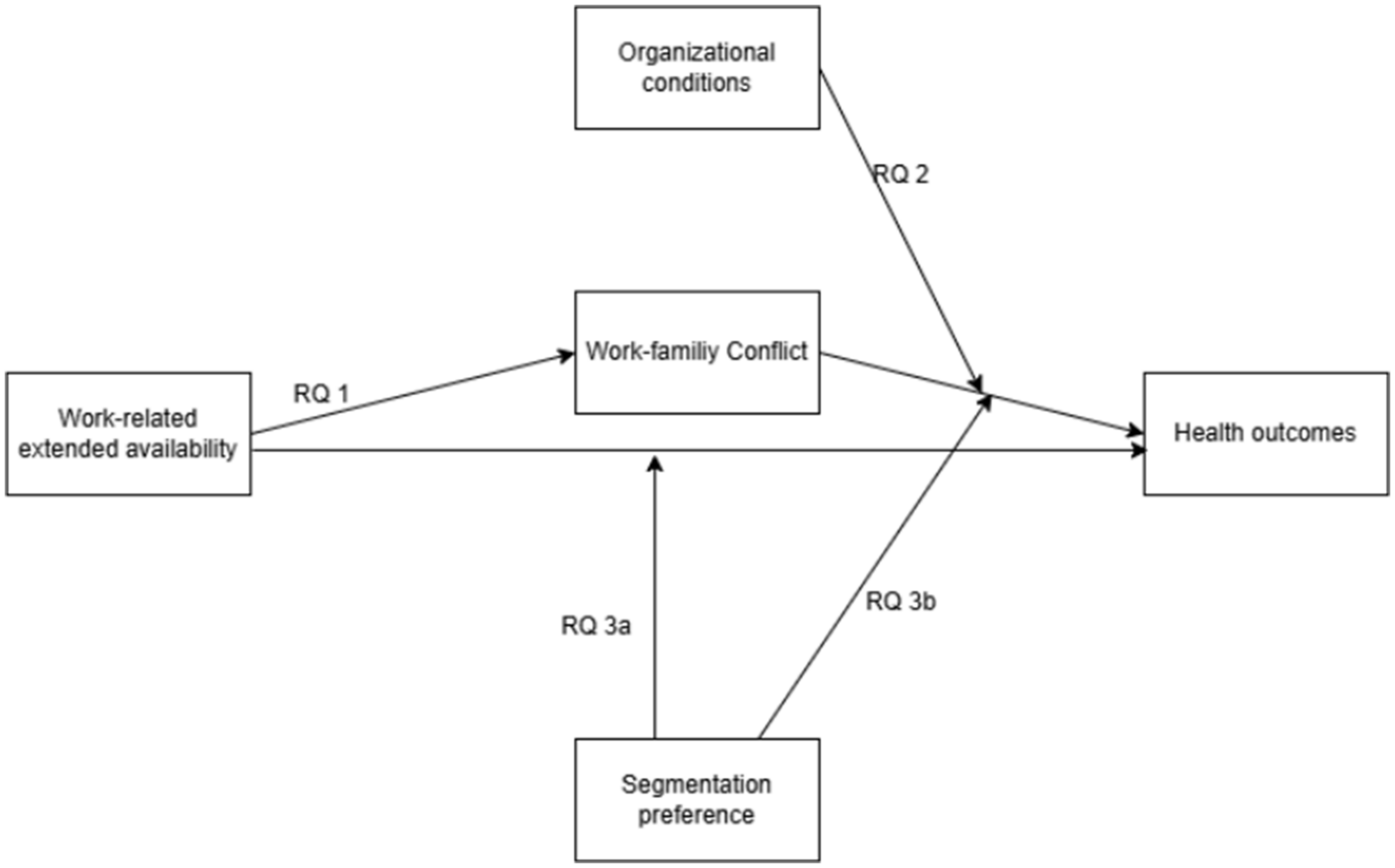
Proposed relationships between extended work-related availability, work-family conflict, health outcomes, and the moderating roles of segmentation preferences and organizational conditions.

The current review will synthesize existing knowledge on work-related extended availability and its impacts on employee well-being, focusing on the influence of moderating and mediating effects. The JD-R Model (Bakker et al., 2023), Work-Life Border Theory (Clark, 2000), and Conservation of Resources Theory (Buchwald and Hobfoll, 2004) provide the framework for a comprehensive understanding of the mechanisms and conditions under which work-related extended availability affects employees with regard to work-family conflict and well-being. Within this conceptual framework, Figure 1 depicts the hypothetical relationships (RQ1-RQ3) between work-related extended availability, work-family conflict, health outcomes, and the moderating variables (i.e., organizational conditions, segmentation preference). In the following, we derive three research questions. First, loss of resources, such as recovery time or emotional energy, may lead to increased stress, burnout, and overall poorer health outcomes (Demerouti et al., 2001; Hobfoll and Shirom, 2001; Maslach et al., 2001; Sonnentag and Fritz, 2007). When employees are continually available, they may experience resource depletion, which manifests as higher stress (Hu et al., 2024), job strain (Hu et al., 2024), and reduced mental well-being (Tedone, 2022). The empirical research (Hu et al., 2024) demonstrates that work-related extended availability depletes personal resources and impairs recovery (as posited by the Conservation of Resources Theory), leading directly to emotional exhaustion and poorer health outcomes (Figure 1, direct effect of work-related extended availability on health outcomes). Studies provide evidence that work connectivity behavior after hours increases emotional exhaustion, while psychological detachment and an individual’s preference for work-family segmentation moderate this effect (Dettmers, 2017; Hu et al., 2024). Work-related extended availability often intrudes on personal and family time, creating a conflict between work and family demands (Allen et al., 2014). Thus, we assume (Figure 1, RQ 1) that higher levels of work-related extended availability increase work-family conflict, which then negatively affects health outcomes (Amstad et al., 2011). In essence, blurred boundaries increase the tension between professional and personal roles. This notion is grounded in boundary theories and supported by empirical findings. For example, studies have shown that when employees have difficulty separating work from home life, they are more likely to experience role overload and conflict, which has been linked to adverse health effects such as emotional exhaustion and burnout (Allen et al., 2014; Kossek and Lautsch, 2012). Moreover, the Conservation of Resources framework posits that when incessant work demands erode recovery time and personal energy, it heightens work-family conflict and accelerates a loss spiral that undermines overall well-being (Hobfoll, 1989). The first research question addresses the mediating effect of work-family conflict in the relationship between work-related extended availability and health outcomes (Figure 1, RQ1):

*RQ1*: Do higher levels of work-related extended availability lead to an increase in work-family conflict, negatively affecting health outcomes?

Second, organizational conditions like job autonomy or social support may act as buffers or protective factors. From the perspective of the JD-R Model (Demerouti et al., 2001; Schaufeli and Taris, 2014), organizational resources may play a significant role in mitigating the negative effects of work-related extended availability on emotional exhaustion. Employees with greater autonomy are likely to regulate their work pace and disconnect when needed, which mitigates emotional exhaustion (van Den Broeck et al., 2010). Employees with greater autonomy experience less exhaustion and better health outcomes. Flexible work arrangements may enable employees to better align work demands with family responsibilities, reducing work-family conflict. This means that even with high work-related extended availability, supportive organizational measures can help protect employees’ health outcomes by reducing work-family conflicts. For instance, Wang (2024) shows that a work-family supportive culture, defined by supervisor support, low career penalties, and low organizational time demands, is associated with lower overall work-family conflict (Wang, 2024). The role of organizational factors, such as structural autonomy and workload control, in mitigating emotional exhaustion and work-family conflict is significant in the relationship between work-related extended availability, work-family conflict and health outcomes, too. For instance, enhanced job autonomy and supportive workplace flexibility may buffer the negative effects of work-related extended availability on work-family conflict. According to Brauchli et al. (2015), who developed the JD-R health model as an extension of the initial JD-R model (Demerouti et al., 2001; Schaufeli and Taris, 2014), job resources are associated with positive biopsychosocial health outcomes. Therefore, the second research question addresses the moderating effect of organizational conditions on the relationship between work-related extended availability, work-family conflict, and health outcomes (Figure 1, RQ2):

*RQ2*: Is the indirect effect of work-related extended availability on health outcomes via work-family conflict moderated by organizational conditions? We expect that higher structural autonomy and workplace flexibility buffer the negative impact of work-family conflict on health outcomes.

Third, individuals differ in their ability to set boundaries between work and non-work roles. For instance, those with strong segmentation preferences or a high capacity for detachment are more likely to limit the spillover effects of work-related extended availability. Focusing on individual differences, the study of Rothbard et al. (2005) examined how personal boundary preferences affect the management of work and family roles. The primary research question referred to how factors such as personality traits, career ambitions, and family responsibilities determine whether a person prefers to integrate or segment their work and non-work activities in life. The researchers prove that these preferences influence individuals’ strategies and significantly affect outcomes such as work-family conflict and overall well-being. Therefore, linking boundary management choices to measurable consequences reinforces the practical relevance of understanding and applying work-life balance concepts. Additionally, the research suggests that employees with strong segmentation preferences or high psychological detachment experience less emotional exhaustion and reduced work-family conflict, thereby reporting better health outcomes (Kossek and Lautsch, 2012; Kreiner et al., 2009; Sonnentag and Bayer, 2005). Employees who prefer clear separations between work and life are less likely to experience stress, as they actively manage the encroachment of work into their personal time. This buffering effect would suggest that personal boundary management moderates the impact of work-related extended availability on work-family conflict and subsequent health outcomes. Similarly, individuals with strong psychological detachment can prevent work-related demands from interfering with family and personal life, thereby reducing health outcomes. Therefore, two final research sub-questions were developed (Figure 1, RQ3a and RQ3b):

*RQ3a*: Moderated direct-effect sub-question: Is the work-related extended availability’s effect on employees’ health outcomes strengthened or weakened by employees’ segmentation preferences?

*RQ3b*: Indirect-effect (moderated-mediation) sub-question: Does work-related extended availability influence employees’ health outcomes indirectly through work-family conflict, and is the strength of this mediated pathway contingent on employees’ segmentation preferences?

These questions are insofar relevant to be addressed with a systematic review as they deepen the understanding of the mechanisms and conditions under which work-related extended availability affects well-being in the workforce in general. This review aims to contribute to developing a more nuanced and comprehensive understanding of work-related extended availability impact on employee well-being and work-family conflict, and to pave the way for investigating the specific cause-and-effect relationship in HROs.

## 2 Research methodology

### 2.1 Systematic review approach

**Figure 2:**
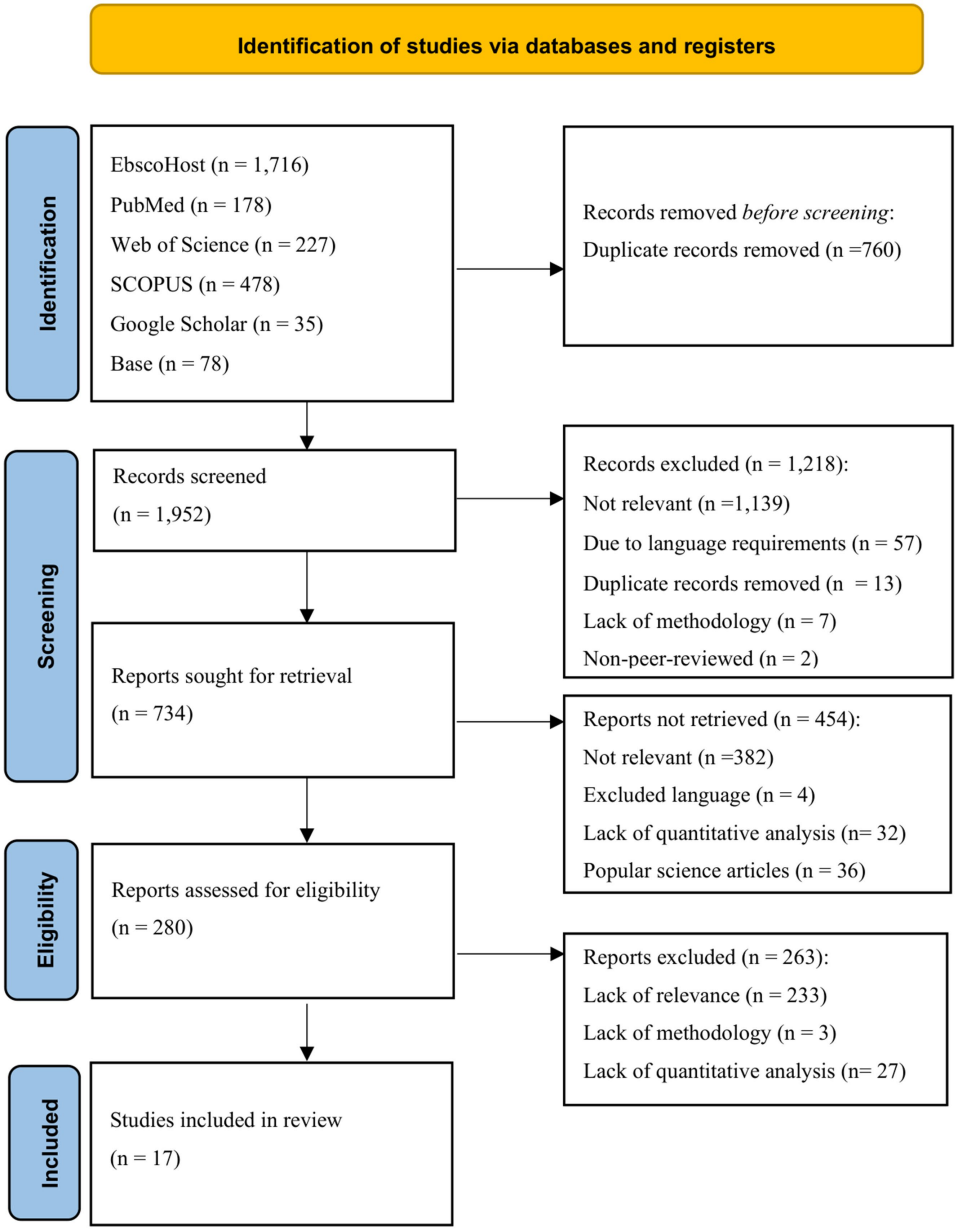
PRISMA 2020 Flow diagram.

The systematic literature review served as the methodological framework for answering the three previously stated research questions (RQ1, RQ2, and RQ3). To ensure methodological transparency, the research protocol was pre-registered on the platform aspredicted.org (ID #215989; https://aspredicted.org/3dsf-wpj3.pdf) before conducting the literature search. The review process strictly adhered to the Preferred Reporting Items for Systematic Reviews and Meta-Analyses (PRISMA) guidelines (Page et al., 2021), following the four sequential phases: identification, screening, eligibility assessment, and inclusion (Figure 2). This structured approach ensured a comprehensive and unbiased selection of relevant studies while maintaining methodological consistency throughout the review process. The literature search was conducted between March 6, 2025, and March 22, 2025. The results of the searches are presented below. The lead author conducted the literature searches. The screening process was also reviewed by a second, independent researcher.

### 2.2 Inclusion and exclusion criteria

Studies were selected based on predefined inclusion and exclusion criteria to ensure relevance to the research questions. The inclusion criteria were as follows:

- Population: employees in organizational and occupational settings exposed to work-related extended availability, constant connectivity, work-related information and communication technology use after hours
- Intervention and Outcome: Original research data (via survey and diary) on work-related extended availability or related constructs (e.g., constant connectivity, work-related information and communication technology use) in relation to health outcomes and/or work-family conflict
- Study design: Only original research papers with quantitative and qualitative (cross-sectional, longitudinal, experimental)
- Time frame: Studies published between 2010 and 2025, with an emphasis on more recent literature
- Publication type: Peer-reviewed journal articles, conference proceedings, and dissertations
- Language: Studies published in English or German

Exclusion criteria included studies focusing solely on technological aspects without addressing psychological or health implications, studies examining general technology use without a specific focus on work-related availability, theoretical papers without empirical data, book reviews, commentaries, and opinion pieces.

Following the PICOS framework, this review focuses on employees in organizational and occupational settings (Population) exposed to work-related extended availability, availability expectations and after-hours connectivity (Intervention/Exposure), compared to employees or groups with lower or no extended availability (Comparators), to identify indicators of work-family conflict, emotional exhaustion, and health-related outcomes (Outcomes) with peer-reviewed quantitative studies published between 2010 and 2025 (Study Designs).

### 2.3 Identification of studies

Figure 2 depicts the whole process of the identification of studies via databases and registers until the final inclusion of studies. The literature was identified from multiple electronic databases to capture a broad spectrum of relevant studies. The databases were strategically selected to encompass various disciplinary perspectives:

- EBSCOhost: Primary source for psychological literature
- PubMed: For health-related research
- Web of Science: For multidisciplinary scientific content
- Scopus: For social science literature
- BASE: For accessing open-access academic resources
- Google Scholar: For capturing additional relevant studies

The search strategy employed a comprehensive set of keywords related to work-related extended availability, work-related communication, health outcomes, and work-family dynamics. These terms were combined using Boolean operators (AND, OR) to create search strings tailored to each database’s specific search mechanisms. For databases with different search capabilities (particularly Google Scholar), the search string was modified while maintaining the same core keywords to ensure optimal search results without compromising comprehensiveness.

The search identified 2,712 records across the databases (EbscoHost: *n* = 1,716, PubMed: *n* = 178, Web of Science: *n* = 227, SCOPUS: *n* = 478, Google Scholar: *n* = 35, Base: *n* = 78). A total of 760 duplicate records were removed before screening, so that 1,952 records were included in the screening process.

The study selection process was designed to ensure thoroughness and minimize bias. It began with an initial screening, during which two researchers independently evaluated the titles and abstracts of all identified records in accordance with pre-established inclusion and exclusion criteria. Studies that satisfied these initial conditions underwent a full-text assessment by the same reviewers. Any discrepancies during the selection process were discussed until a consensus was reached. Throughout the review process, using Zotero (Version 7.0.11) as the reference management software played an essential role, facilitating the organized categorization and efficient retrieval of studies during the various phases of the review. During the title screening, a total of 1952 records were reviewed. A total of 1,218 records were excluded. Of these, 1,139 records were excluded based on the irrelevance of the addressed topics. An additional 57 records were excluded because they were published in another language (i.e., French, Spanish, Dutch, and Russian) than English or German. Thirteen duplicates were identified, seven studies were excluded due to lack of methodology (non-empirical works), and two more studies were non-peer-reviewed publications. The remaining 734 records were classified as potentially relevant and evaluated in the next step, the abstract screening.

Initial trends suggest that structural aspects of organizations are less frequently addressed, but there are numerous studies on extended reachability, particularly in connection with work-family conflicts. In this step, an additional 382 studies were excluded due to lack of relevance. Four studies were published in a language other than English or German, and 32 studies were not statistically evaluated. Furthermore, 36 popular science articles were found among the search results and were also excluded. This left 280 studies for full-text screening.

During the full-text screening, 233 studies were excluded due to lack of relevance, three due to lack of methodology, and 27 due to lack of quantitative statistical evaluation. After the full-text evaluation, 17 studies remained for the literature review. Figure 2 visualizes the detailed selection process. According to article screening and excluding articles based on the above criteria, following 17 studies were included in the final review: Adams and Schwarz (2024), Allgood et al. (2022), Bauwens et al. (2020) Bayazit and Bayazit (2019), Becker and Lanzl (2023), Cho et al. (2020), Choi et al. (2022), Derks et al. (2016), Elshaer et al. (2024), Gadeyne et al. (2018), Haar and Wilkinson (2023), Knardahl and Christensen (2022), Martineau and Trottier (2023), Schieman and Young (2013), Stempel et al. (2022), Thörel et al. (2021), and Wright et al. (2014). Supplementary Table S1 provides an overview of the key features of the included studies.

### 2.4 Data extraction and quality assessment

For each included study, relevant data were systematically extracted using a standardized form. This form captured comprehensive study characteristics, including authors, publication year, country, study design, sample size, and population details. Methodological features were also documented, encompassing measurement instruments, analytical approaches, and key variables examined. The extraction process carefully recorded the main findings, including statistical results, effect sizes, and qualitative insights that emerged from the research. Additionally, underlying theoretical frameworks and conceptual models that informed each study were noted. Subsequently, the bias assessment was conducted using the ROBINS-I framework, which is particularly suited for evaluating non-randomized studies. This study systematically examined each research article across various domains, including confounding, exposure measurement, participant selection, post-exposure interventions, missing data, outcome measurement, and selective reporting. The risk levels were mapped to corresponding ROBINS-I categories: “Low,” “Moderate” and “Serious” to ensure consistency in interpretation. Using the robvis package in R (McGuinness and Higgins, 2020), a rating system (see Table 1) was generated to provide an overarching view of the risk-of-bias assessments, illustrating the proportion of studies falling into each risk category across the evaluated domains:

- For the risk of bias due to confounding (Domain 1), studies were evaluated based on their design and analytical approach. Longitudinal, panel, or cross-lagged designs that explicitly mentioned adjustment for confounders received “Low” risk ratings, while cross-sectional designs without clear confounder control were assigned “Moderate.”
- The risk of bias in the selection of participants (Domain 2) considered sample characteristics, with large representative samples receiving “Low” risk ratings, very small samples (less than 50 participants) rated “High” risk, and intermediate sample sizes (at least 50 participants) assigned “Moderate.” Adequate power reduces the chance of selecting an unrepresentative subset purely by chance. For most prevalence or mean-difference questions, a minimum of approximately 80% power requires roughly 64 participants per group when detecting a medium effect size (*d = 0.50, α = 0.05*) (Cohen, 1988). Consequently, studies with fewer than 50 total participants are unlikely to meet conventional power standards and are rated “High” risk. The sampling strategy was also relevant, while non-probability samples were evaluated as at least “Moderate.”
- The risk of bias due to the classification of the intervention (Domain 3) was assessed based on the type of measurement used. Studies that used validated or objective measurements were classified as “Low” risk, while studies that relied solely on subjective or self-report methods were classified as “Moderate” risk.
- For risk of bias due to post-exposure interventions (Domain 4), the evaluation examined whether any interventions after baseline were controlled or randomized, with such cases rated “Low” risk. In the absence of relevant interventions, a default “Low” rating was applied.
- The risk of bias due to missing data (Domain 5) assessed how studies handled incomplete data. Studies reporting appropriate missing data handling methods received “Low” risk ratings, while those with unaddressed dropout or attrition issues were assigned “Moderate.” When no details were reported, a “Low” rating was assumed.
- For the risk of bias arising from the outcome measurement (Domain 6), studies using validated and established scales were rated as “Low” risk. In contrast, scales without prior validation were assigned as “Moderate.”
- The risk of bias in selecting the reported result (Domain 7) evaluated whether studies selectively reported outcomes or analyses that yielded significant or favorable results.

**Table 1:** Risk of bias assessment for included studies (*N* = 17) using the ROBINS-I tool (low = low risk of bias; moderate = moderate risk of bias).

Article listing by researcher	Risk of bias due to						
	Confounding	Participant selection	Intervention classification	Deviations from intended interventions	Missing data	Outcome measurement	Selective reporting
Adams and Schwarz (2024)	Low	Low	Low	Low	Low	Low	Low
Allgood et al. (2022)	Moderate	Moderate	Low	Low	Low	Low	Low
Bauwens et al. (2020)	Moderate	Moderate	Low	Low	Low	Low	Low
Bayazit and Bayazit (2019)	Moderate	Moderate	Low	Low	Low	Low	Low
Becker and Lanzl (2023)	Moderate	Moderate	Low	Low	Low	Low	Low
Choi et al. (2022)	Moderate	Moderate	Low	Low	Low	Low	Low
Cho et al. (2020)	Moderate	Moderate	Low	Low	Low	Low	Low
Derks et al. (2016)	Moderate	Moderate	Low	Low	Low	Low	Low
Elshaer et al. (2024)	Moderate	Moderate	Low	Low	Low	Low	Low
Gadeyne et al. (2018)	Moderate	Moderate	Low	Low	Low	Low	Low
Haar and Wilkinson (2023)	Low	Low	Low	Low	Low	Low	Low
Knardahl and Christensen (2022)	Moderate	Moderate	Low	Low	Low	Low	Low
Martineau and Trottier (2023)	Moderate	Moderate	Low	Low	Low	Low	Low
Schieman and Young (2013)	Moderate	Moderate	Low	Low	Low	Low	Low
Stempel et al. (2022)	Moderate	Moderate	Low	Low	Low	Low	Low
Thörel et al. (2021)	Low	Low	Low	Low	Low	Low	Low
Wright et al. (2014)	Low	Low	Low	Low	Low	Low	Low

Table 1 depicts the risk of bias assessment for the included studies. The seventeen studies that form the evidence base differ little in their measurement practices. For each study, the methodology relied on validated scales or server logs. Hence, bias in classification of the exposure (Domain 3) and in outcome measurement (Domain 6) is uniformly low, but they diverge markedly in design and sampling, which ultimately determines the overall risk-of-bias label. Allgood et al. (2022), Bauwens et al. (2020), Elshaer et al. (2024), Becker and Lanzl (2023), Stempel et al. (2022), and the daily-diary studies by Cho et al. (2020) and Derks et al. (2016) are typical of the first wave of work: each study relies on either a single-wave survey or a short (ten-day and four-day) diary administered to convenience volunteers. Because temporal precedence cannot be demonstrated and self-selection may filter out employees who deliberately avoid after-hours information and communication technology, these six studies incur moderate bias due to confounding (Domain 1) and participant selection (Domain 2). No problematic deviations from intended interventions are evident (Domain 4), missing data is either minimal or correctly handled (for instance by using advanced imputation techniques, such as multiple imputation, Domain 5), and the authors report all prespecified outcomes (Domain 7), so the worst remaining domain and therefore the overall rating stays at “Moderate.” Studies conducted by Bayazit and Bayazit (2019), Gadeyne et al. (2018), Martineau and Trottier (2023), Wright et al. (2014), and Schieman and Young (2013) also adopt similar single-wave strategies. As in the earlier group, robust instrumentation holds measurement bias low, yet cross-sectional timing and non-probability recruitment keep confounding and selection risks moderate; consequently, each receives a moderate overall judgment. By contrast, Choi et al. (2022) draw on the nationally stratified Korean Working Conditions Survey, which lowers selection bias to a low level. Nevertheless, their one-time assessment of both technology use and work-family conflict retains moderate confounding, so the overall assessment remains moderate. Similarly, Knardahl and Christensen (2022) analyzed data from 13,119 employees from various organizations, 5,228 of whom provided information about their expectations regarding availability. The combination of cross-sectional and prospective analyses ensured validated exposure and outcome measures, but voluntary participation and attrition led to moderate risks of bias and participant selection. Missing data were transparently reported and appropriately handled, and no deviations or selective reporting were identified. Overall, the study received a moderate rating for bias potential, mainly due to self-selection and residual bias.

By contrast, Adams and Schwarz (2024) use a nationally stratified random sample from the population register, apply validated multi-item measures for all constructs with high reliability, and explicitly consider important demographic and occupational confounding factors in all models. Furthermore, they report no deviations from the intended procedures, treat minimal missing data using multiple imputation, and present all pre-specified results and analyses without selective reporting. Thörel et al. (2021) break new ground with a three-wave cross-lagged panel. The longitudinal structure largely resolves confounding, earning an overall low rating. Haar and Wilkinson (2023), using a nationally representative New Zealand workforce panel with multiple-imputation handling of dropout, manage to combine probability sampling, longitudinal data, and pre-registered analyses with confounding, selection, missing data, and reporting all rated low, and measurement bias already low. The studies by Haar and Wilkinson (2023), Thörel et al. (2021), and Adams and Schwarz (2024) meet every ROBINS-I threshold and receive a low overall judgment. The synthesis, therefore, retains every study but applies sensitivity analyses to confirm that removing the median-sized moderate-quality contributions does not materially shift the pooled estimates.

## 3 Results

### 3.1 Research question 1

The first research question “Do higher levels of work-related extended availability lead to an increase in work-family conflict, negatively affecting health outcomes?” refers to the mediation pathway, assuming that work-related extended availability increases work-family conflict, and that, as a consequence, would lead to negative health outcomes (Figure 1, RQ 1). To answer the first research question, we included seven studies (Adams and Schwarz, 2024; Choi et al., 2022; Derks et al., 2016; Elshaer et al., 2024; Haar and Wilkinson, 2023; Knardahl and Christensen, 2022; Wright et al., 2014) that addressed the topic in their research and report their main results concerning RQ 1 in more detail. Supplementary Table S1 provides an overview of the study’s key features.

The study by Adams and Schwarz (2024) adopts a holistic approach that examines how flexible work arrangements impact work-family conflict. This research examines work-family conflict guided by work/family border theory and the flexibility paradox, using data from the German Family Panel survey, which comprised 1,983 participants who were almost evenly distributed in terms of the number of female and male participants. This study’s analytical process can be outlined in three steps: first, a mediation analysis is utilized to examine the effect of working from home on work-family conflict; second, the effect of work demands on the mediation path was analyzed; and third, the moderated mediation was undertaken separately for mothers and fathers to determine if a gender difference was present. Results reveal that working from home was associated with increased work-family conflict stemming from unclear boundaries between work and family domains, demonstrating the factor of digitally extended availability as a mediator and the working demand as a moderator. Finally, the interaction effect between digitally extended availability and work demands is significantly associated with work-family conflict for fathers but not for mothers.

Choi et al. (2022) examine the association between work-related communication technology used after working hours and work-family conflict during the COVID-19 pandemic using data from the 6th Korean Working Conditions Survey (KWCS). The KWCS study has the benefit of utilizing primary data from a reported dataset that includes a large sample of 17,426 waged workers with a spouse and at least one child. The methodology employs a logistic regression analysis to examine the association between work-family conflict and work-related communication technology used after regular working hours, while controlling for important covariates such as gender and work hours. Two main steps are taken in the analysis. The first step involves logistic regression analysis to generate the odds ratios associated with communication technology for work-family conflict. The second step involved stratifying the odds ratios for work-family conflict by gender and working hours to determine if there are differential effects by these groups. The results showed that work-related communication technology used outside work hours was significantly associated with increases in work-family conflict, and more so for women than for men. The subgroup of women who worked more than 52 h a week had the highest odds ratio for work-family conflict.

Derks et al. (2016) conducted a four-day diary study with 71 employees to investigate the effects of smartphone use outside of work on work-family conflict. They found that such use was generally associated with higher levels of work-family conflict, but the preference for segmentation mitigated this association: among integrators, smartphone use outside of work hours was associated with lower levels of work-family conflict, while no significant effect was observed among segmenters.

Elshaer et al. (2024) examined work-related mobile internet use during off-job time and its association with quality of life. They used structural equation modeling on data from 341 faculty members in Egypt’s tourism and hospitality faculties and examined their hypothesized model using partial least squares. The results indicated that work-related smartphone use during off-job hours negatively impacted quality of life, with work-family conflict as a key mediator in the relationship. The emerging evidence supports the hypothesis that problems managing work and family responsibilities influence overtime activities, running a household, and parent–child relationships, which can negatively affect the family’s quality of life.

Knardahl and Christensen (2022) analyzed cross-sectional and prospective data to examine the effects of working from home and expectations regarding availability outside regular working hours. While 13,119 employees provided information about their working hours at home, only those who worked from home were asked about expectations regarding their availability, resulting in an analytical sample of 5,228 employees (3,146 cross-sectional and 2,082 prospective data). The study used validated survey measures of working conditions, well-being, and organizational factors, enabling a robust assessment of both work demands and health-related outcomes. The results showed that expectations regarding availability were associated with higher work demands, role conflicts, work-family conflicts, psychological stress, sleep problems, and lower levels of support from colleagues and supervisors. Importantly, these effects were only observed in the cross-sectional analyses, while no prospective associations were found. Overall, the study highlights the immediate but not long-term effects of extended availability on employee health outcomes.

Haar and Wilkinson's (2023) analyzed representative data from 422 New Zealand employees across various jobs, sectors, and industries, provided in late 2020. They explored how mobile work and work-related extended availability impacts employees of various ages. Work-related extended availability was measured using three items each for work-family conflict and family–work conflict, and an additional item was included for contextual evidence of COVID-19. The substantial focus was on job anxiety, job depression, and insomnia. The methodology included a confirmatory factor analysis and a moderated mediation model. The findings suggested that employees aged 55 years or older experienced greater negative repercussions of work-related extended availability than younger employees, with mobile work positively impacting both work-family and family-work conflicts. All health outcomes (job anxiety, job depression, and insomnia) were significantly associated with mobile work in the expected adverse direction, with work-family conflict and family-work conflict operating as mediators, and age interacting with mobile work. Specifically, age moderated these associations such that effects were stronger at higher age. The study did not determine whether this pattern reflects chronological age per se, family composition, or greater role responsibility. The study established that age functions as a boundary condition, and the indirect effect of mobile work on employee well-being outcomes increased with age. The analysis of the behavioral risk of mobile work using a mobile device for work usage when spending time with family, and associated burdens for older workers, adds to the importance of examining these employee-focused practices.

Wright et al.’s (2014) study examined the relationship between using work-related communication technology outside traditional work hours and employee perceptions of work-life conflict, burnout, turnover intentions, and job satisfaction. The study used an online survey to collect data from 168 employees of over 30 companies in a Midwestern city. The results showed a significant relationship between the number of hours spent using work-related communication technologies outside traditional work hours and employee perceptions of work-life conflict, confirming and supporting past research on the negative effects of after-hours connectivity. Results further demonstrated that attitudes toward communication technologies significantly moderated the relationships among the studied outcomes. Those with positive attitudes towards using communication technologies reported decreased work-life conflict. This suggests that individual attitudes and preferences regarding the use of a technology may buffer against negative outcomes. After controlling for employee age, perceived stress in life, and attitudes towards communication technologies, work-life conflict predicted recovery from job burnout and job satisfaction but did not predict turnover intentions.

Overall, the seven studies that explicitly address RQ1 consistently show that extended availability via information and communication technology outside regular working hours exacerbates the work-family conflict and thus impairs health (Adams and Schwarz, 2024; Choi et al., 2022; Derks et al., 2016; Elshaer et al., 2024; Haar and Wilkinson, 2023; Knardahl and Christensen, 2022; Wright et al., 2014). Five of the seven studies (Adams and Schwarz, 2024; Choi et al., 2022; Derks et al., 2016; Knardahl and Christensen, 2022; Wright et al., 2014) explicitly confirm that the use of technology after work is significantly associated with a higher level of conflict between work and family life. For example, Adams and Schwarz (2024) show that flexible working arrangements in an “always-on” culture blur the boundaries between work and private life, thereby increasing work-family conflicts. Similarly, Derks et al. (2016) show that the effects of work-related smartphone use depend on the style of boundary management: among integrators, such use was associated with lower levels of conflict, while no effects were observed among segmenters. Two studies (Elshaer et al., 2024; Haar and Wilkinson, 2023) also show that work-family conflicts mediate the effects of extended availability on health outcomes such as job anxiety, job depression, and insomnia. In addition, three studies (Adams and Schwarz, 2024; Choi et al., 2022; Wright et al., 2014) highlight subgroup-specific vulnerabilities: fathers in high-demand jobs show a stronger indirect effect from working from home (via work-related extended availability) on work-family conflict, women who work more than 52 h per week experience increased conflict, and older workers show stronger indirect effects of mobile work on health outcomes. Taken together, all seven studies conclude that work-related extended availability has a lasting negative impact on employee health outcome through the central mechanism of work-family conflict, with several studies additionally identifying subgroup-specific vulnerabilities.

### 3.2 Research question 2

The previously discussed path from work-related extended availability to work-family conflict to health outcomes can be subject to various moderating influences, in particular, characteristics of job design. Building on this assumption, the second research question asks: “Do job design characteristics and organizational resources influence the relationship between extended availability at work, work-family conflict, and health outcomes?” and refers to the moderation path, assuming that contextual and individual resources can cushion or amplify the negative effects of extended availability on work-family conflict and the resulting health outcome (Figure 1, RQ 2). To answer the second research question, we included six studies (Allgood et al., 2022; Bayazit and Bayazit, 2019; Cho et al., 2020; Gadeyne et al., 2018; Schieman and Young, 2013; Stempel et al., 2022) that explicitly addressed moderating influences. These included supportive leadership, family-friendly corporate cultures, social belonging, and individual boundary management, which helped to mitigate the daily demands associated with information and communication technology and reduce negative effects. Supplementary Table S1 provides an overview of the study’s key features.

The cross-sectional study by Bayazit and Bayazit (2019), where survey data were collected from 213 professionals in Istanbul, Turkey, demonstrated that flexibility, in terms of individualized arrangements (i-deals), mediate the relationship between flexible work arrangements and work-family conflict. Moreover, family-supportive cultures moderate the relationship between individualized arrangements and family-work conflict, suggesting that organizational culture influences how flexibility arrangements affect the work-family conflict. Supervisory behaviors significantly moderate both stages of this pathway.

Stempel et al. (2022) conducted a cross-sectional employee survey linking work-related extended availability, work-family conflict, work performance, and work-life-friendly supervisor role modeling. The online survey included 258 employed psychology students in Germany (43% female). The findings revealed that work-life-friendly role modeling by supervisors attenuates the detrimental indirect effect of work-related extended availability on performance via work-family conflict: when supervisors model healthy boundary management, employees report less work-family conflict despite work-related extended availability, and the indirect path to reduced performance is weakened. Social resources also moderate the indirect path from work-related extended availability to health outcomes via work-family conflict, as shown for example by Allgood et al. (2022). Allgood et al.’s (2022) study investigates the impact of social belonging and instrumental leadership on work-family conflict and burnout among employees, particularly during the COVID-19 pandemic. The research relies on cross-sectional survey data collected from local government employees in a large city in the southwestern United States, with a population exceeding 500,000. The survey data were gathered in May 2020. Out of an initial set of 241 contacts, 124 employees provided complete responses, resulting in a response rate of 51%. The respondents were almost evenly split by gender (50.8% female). The study is grounded in the JD-R model, proposing that organizational resources such as instrumental leadership (a vertical resource) and a sense of social belonging (a horizontal resource) can help mitigate burnout by alleviating conflicts between work and family-life activities. Findings showed that workers who feel a strong sense of social belonging respond to work-related extended availability with less work-family conflict and, as a result, report lower levels of burnout. The findings highlight the importance of horizontal organizational resources during periods of disruption (i.e., COVID-19 pandemic). By contrast, instrumental leadership showed only marginal direct associations with work-family conflict and burnout and no significant indirect effect via work-family conflict. The study also explores the relationship between work-family conflict and burnout, noting a strong correlation, indicating that these constructs share approximately half of their variance. This close link underscores the significant interplay between work-family conflict and burnout, emphasizing the critical role of social belonging in reducing burnout. The study concludes that social belonging, as a horizontal organizational resource, is more crucial for reducing burnout during periods of disruption than the more formal, vertical resource of instrumental leadership.

Cho et al.’s (2020) study examines how daily information and communication technology demands contribute to work-family conflict on a day-by-day basis. The researchers conducted a full daily diary study of 98 full-time employees over 10 workdays, for 793 day-level observations. This method allowed researchers to examine how information and communication technology demands experienced during working hours and time afterward contribute to work-family conflict in the evening. The study examined the timing and nature of information and communication technology demands and their effect on work-family processes, and importantly, accounts for individual boundaries. The research follows two main analyses. First, a multilevel mediation model was calculated to test the negative spillover effect of on-the-job information and communication technology demands on evening work-family conflict through the negative impact by the end of the workday. The findings showed that the spillover of information and communication technology demands from work into the non-work domain increases end-of-work negative affect in employees which then spills over into higher work-family conflict. Second, a multilevel path analysis estimated the impact of off-the-job information and communication technology demands on work-family conflict and tested a cross-level moderation by boundary control. Results indicated that higher work-related extended availability was associated with higher evening work-family conflict. This association was weaker among employees reporting higher boundary control. Furthermore, the study tests the buffering effect of boundary control for these relationships. The findings showed that those who perceive a high amount of boundary control reported a weaker relationship between off-the-job information and communication technology demands and work-family conflict, implying that people’s ability to control boundaries between their work and personal lives can lessen the negative effects of information and communication technology demands on their work-family balance.

The study by Schieman and Young (2013) used data from the 2011 Canadian Work, Stress, and Health Study, a large national sample of 5,729 working adults. The main goal of this study was to examine whether work-related extended availability is related to psychological distress and work-family conflict. The results showed that work-related extended availability is associated with higher levels of work-family conflict, psychological distress, and sleep problems. These results emphasize the potential negative consequences of after-hours work communications for the well-being of employees.

Gadeyne et al. (2018) investigate the connection between work-related information and communication technology use outside of work hours and work-home conflict (including work-family and non-family private life). The data utilized a survey of 467 working parents in Belgium. Gadeyne et al. (2018) explored three-way interactions among two types of work-related information and communication technology use outside of work (i.e., smartphone use and PC/laptop use), integration preference, and two work environment characteristics (organizational integration norms and work demands) on time- and strain-based work-to-home conflict. The results indicate that only work-related PC/laptop use outside work hours relates positively to work-home conflict (not smartphone use). Work-related PC/laptop use is buffered when integration preference exists, but only if the work environment exhibits low organizational integration norms or low work demands. This means that when individuals have an integration preference, work-related information and communication technology use outside of working hours may not complicate work and home life balance and may even facilitate it.

The six studies addressing RQ2 (Allgood et al., 2022; Bayazit and Bayazit, 2019; Cho et al., 2020; Gadeyne et al., 2018; Schieman and Young, 2013; Stempel et al., 2022) consistently show that contextual and individual moderators influence the relationship between work-related extended availability, work-family conflict, and health outcomes. Four studies (Allgood et al., 2022; Bayazit and Bayazit, 2019; Schieman and Young, 2013; Stempel et al., 2022) identified organizational resources, such as supportive leadership, family-friendly cultures, or social belonging, as important buffers that mitigate the negative effects of work-related extended availability on work-family conflict and the negative health outcomes. For example, Bayazit and Bayazit (2019) showed that family-friendly corporate cultures mitigate the effects of flexibility individualized arrangements on work-family conflict, while Stempel et al. (2022) found that supervisors modeling healthy boundaries reduce the indirect effect of work-related extended availability demands on performance through work-family conflict. Two studies (Cho et al., 2020; Gadeyne et al., 2018) emphasized moderators at the individual level, such as boundary control and integration preferences, which moderated the extent to which daily information and communication technology demands affected family life. At the individual level, Cho et al. (2020) reported that employees with higher boundary control experienced a weaker association between daily information and communication technology demands and work-family conflict in the evening, and Gadeyne et al. (2018) showed that integration preferences can mitigate or even reverse the negative effects of information and communication technology use outside of work, depending on organizational norms and workload. Similarly, Schieman and Young (2013) found that professional autonomy and control over work schedules mitigated the effects of after-hours contact on health outcomes such as psychological distress and sleep problems. Overall, these findings suggest that both organizational and individual resources mitigate the indirect path from work-related extended availability to health outcome via work-family conflict, underscoring the importance of workplace design and boundary management in protecting employee health.

### 3.3 Research questions 3a-b

To answer the research question, “Do employees’ segmentation preferences moderate the effects of work-related extended availability on health outcomes, both directly (Figure 1, RQ3 a) and indirectly via work-family conflict (Figure 1, RQ3 b)?,” we included four studies (Bauwens et al., 2020; Becker and Lanzl, 2023; Martineau and Trottier, 2023; Thörel et al., 2021). Supplementary Table S1 provides an overview of the studies key features.

Martineau and Trottier (2023) studied the effect of job design elements on work-life boundaries and conflict during mandated telework, a ubiquitous situation due to COVID-19. Work-life refers conceptually to a broader context than work-family, and includes non-family private life. The authors sought to determine the relationship between work-life integration and the process whereby boundaries can be enacted to diminish work-life conflict. The study focused on two work design features, autonomy and job feedback, that can function as stressors on work-life balance, thereby influencing work-life conflict. They suggested achieving this by employing boundary enactment strategies that tilt toward segmentation, which can assuage inter-role conflict, even though they do not necessarily mediate outcomes. The study’s sample consisted of 93 employees (82.8% female) of an accounting association, the Quebec CPA Order. Participants were recruited through collaboration with the human resources department, which, on behalf of the researcher, invited all employees to complete a self-administered questionnaire via email in the winter of 2021. The study’s findings show that the relationship between job feedback and a worker’s work-life conflict performance is significantly mediated by boundary enactment, supporting segmentation. This indicates that job feedback has a direct influence on work-life conflict through the mechanism of boundary enactment. The statistical analysis demonstrated that job feedback alone significantly impacts work-life conflict and boundary enactment.

Becker and Lanzl (2023) analyzed associations between technology driven communication demands (i.e., information and communication technology use and demands, interruptions, invasions and overload) and work-family conflict during the COVID-19 pandemic with a longitudinal approach over approximately 6 months during the COVID-19 pandemic using a two-wave longitudinal design. The sample consisted of 637 working adults (41.1% female). Segmentation preference was assessed on a segmentation integration continuum and used as a moderator via multigroup comparison. This study provides a contextual background as remote work has transitioned to the “new normal,” and many people have been working from home because of COVID-19. Specifically, the authors examined whether distinct information and communication technology use and demands and communication channels (e.g., email, video calls) predict work-family conflict and whether these effects differ by segmentation preference. Invasions and overload were positively related to work-family conflict, email use appeared comparatively less intrusive for integrators, and video calls were associated with overload across groups and with invasions, particularly among segmenters. This has caused boundary management and individual preference for segmenting work and personal life to receive greater attention, particularly because digital technologies are intrusive and can exacerbate segmentation issues. Overall, effects on well-being were stronger for segmenters than integrators, primarily supporting RQ 3b (channel-specific moderation by segmentation preference) and, secondarily, RQ 3a (stronger overall effects for segmenters) regarding boundary preferences as moderators of information and communication technology-driven demands and work-family outcomes. Becker and Lanzl (2023) extend the literature on ongoing technology-driven spillover effects and utilizing longitudinal data collected during the pandemic. Practically, the findings suggest that the design and management of digital communication tools and practices should account for individual boundary management preferences. In their analytic strategy, the authors examined whether information and communication technology use and demands can be associated with role conflict. Together, these results underscore the boundary-transcending effects of technology use and inform recommendations for employers developing the “new normal” of remote work.

However, Bauwens et al. (2020) and Thörel et al. (2021) found no evidence that segmentation preferences moderated the relationship between work-family conflict and health outcomes. Bauwens et al. (2020) examined the relationship between teachers’ use of information and communication technology for work tasks outside of formal working hours and how that impacts teachers’ work-life balance. Their research examined how technology acceptance (particularly performance expectancy) relates to work-related use of information and communication technology after hours and work-life balance, and how teachers’ segmentation preferences influence each relationship. The authors collected data from 288 secondary school teachers from Flanders, Belgium, inquiring about their use of a digital learning environment outside the school grounds and school hours. The authors used structural equation modeling to analyze their data and found that social influence negatively impacted teachers’ work-life balance, mediated by the use of information and communication technology outside of formal working hours. The authors did not find support for any of the other technology acceptance factors or the moderating role of integration preference.

Thörel et al. (2021) aimed to elucidate how work-related extended availability may influence health, recognizing individual differences. The authors adopted a three-wave cross-lagged panel design with a sample of 528 working adults. The authors propose that the relationship between work-related extended availability and health outcomes, such as sleep and exhaustion, is mediated by detachment, and that one’s segmentation preference would moderate their relationship. The authors found a cross-lagged negative relationship between work-related extended availability and detachment. However, there was no indirect relationship between work-related extended availability and either sleep or exhaustion through detachment, nor did the authors find evidence of moderation effects of segmentation preferences in the relationship between work-related extended availability and any health outcomes. Overall, the evidence is mixed. For RQ3a, only Thörel et al. (2021) directly tested whether segmentation preferences moderate the link between work-related extended availability and health outcome, and did not find a moderation. Becker and Lanzl (2023) observed moderation by segmentation preference between information and communication technology use and technology-related demands, operationalized as invasions, interruptions, and overload, but not between these demands and work-family conflict. Bauwens et al. (2020) reported no moderation for work-life balance. For RQ3b, none of the four studies directly examined a moderated mediation via work-family conflict. Martineau and Trottier (2023) analyzed boundary enactment mediating work design to work-life conflict; Thörel et al. (2021) examined mediation via psychological detachment and found no indirect effects on health outcomes; Becker and Lanzl (2023) linked information and communication technology related demands to work-family conflict without testing a moderated mediation on that path; and Bauwens et al. (2020) focused on work-related use of information and communication technology after hours and work-life balance without a moderated mediation via work-family conflict. We included them to map the constituent links and boundary conditions of RQ3b, even though the full moderated mediation was not estimated. Taken together, these findings point to a clear evidence gap for RQ3b that we highlight in our synthesis.

## 4 Discussion

The present review synthesized existing knowledge on work-related extended availability and its impact on employee health outcomes in a generic work context. The focus was set on the influence of moderating and mediating effects to deepen the understanding of the relationship between work-related extended availability and health outcomes. In the following, we discuss the results regarding the three research questions and synthesize the identified mediators and moderators (Table 2). We will prioritize the mediation and moderation variables by evidential consistency and proximity to health outcomes.

**Table 2:** Mediators and moderators (prioritized).

Rank	Variable	Role	Representative studies
1	Work–family conflict	Mediator (health outcomes): RQ 1	Adams and Schwarz (2024), Choi et al. (2022), Derks et al. (2016), Elshaer et al. (2024), Haar and Wilkinson (2023), Knardahl and Christensen (2022), and Wright et al. (2014)
2	Family-supportive culture/social belonging	Moderator (organizational): RQ 2	Bayazit and Bayazit (2019), Allgood et al. (2022), and Wang (2024)
3	Supervisor role modeling (healthy boundaries)	Moderator (organizational): RQ 2	Stempel et al. (2022)
4	Job autonomy/schedule control	Moderator (organizational): RQ 2	Schieman and Young (2013)
5	Boundary control	Moderator (individual): RQ 2	Cho et al. (2020)
6	Job pressure (demands)	Moderator (amplifier): RQ 2	Schieman and Young (2013)
7	Technology type & timing (PC/laptop vs. smartphone; after-hours)	Context/conditional moderator: RQ 3a	Gadeyne et al. (2018)
8	Segmentation preferences	Moderator (individual; mixed evidence): RQ 3a	Becker and Lanzl (2023), Bauwens et al. (2020), and Thörel et al. (2021)
9	Subgroup indicators (age; long hours; fathers in high-demand jobs)	Interaction/vulnerability: RQ 2	Haar and Wilkinson (2023), Choi et al. (2022), and Adams and Schwarz (2024)

Seven studies (Adams and Schwarz, 2024; Choi et al., 2022; Derks et al., 2016; Elshaer et al., 2024; Haar and Wilkinson, 2023; Knardahl and Christensen, 2022; Wright et al., 2014) explicitly addressed the research question 1 “Do higher levels of work-related extended availability lead to an increase in work-family conflict, negatively affecting health outcomes?.” Across the analyzed seven studies, work-related extended availability outside regular working hours was generally associated with higher work-family conflict and, in turn, poorer health outcomes. From a theoretical perspective, the findings support a mechanism whereby work-related extended availability elevates work-family conflict and is associated with adverse health outcomes, consistent with conservation-of-resources accounts (Haar and Wilkinson, 2023; Elshaer et al., 2024) and with work-family border perspectives that emphasize boundary permeability under work-related extended availability (Derks et al., 2016; Choi et al., 2022; Adams and Schwarz, 2024; Wright et al., 2014). In addition, evidence on availability expectations situates these effects within boundary norms (Knardahl and Christensen, 2022). Taken together, the analyzed studies support work-family conflict as the most influential and proximal mediator linking work-related extended availability to health outcomes (Table 2). Subgroup analyses indicate heightened vulnerability among fathers in high-demand jobs (Adams and Schwarz, 2024), women working more than 52 h per week (Choi et al., 2022), and older workers (Haar and Wilkinson, 2023). Adams and Schwarz (2024) found that flexible work arrangements, while offering potential benefits, can blur the boundaries between work and personal life, leading to increased work-family conflict. Their study revealed that working from home is associated with heightened work-family conflict due to these blurred boundaries. Similarly, Choi et al. (2022) confirmed that using work-related communication technology outside regular working hours is significantly associated with higher work-family conflict in nationally representative data collected during the COVID-19 pandemic. Since this effect was more pronounced among women, the vulnerability of this subgroup to work-family conflict is particularly evident. Elshaer et al. (2024) further illustrated that work-related information and communication technology usage during off-job time is associated with poorer health outcomes, with work-family conflict as a significant mediator. This study emphasized that the negative effects persist even after controlling for various demographic factors, underscoring the pervasive impact of work-related extended availability on health outcomes. In line with these findings, Knardahl and Christensen (2022) reported that availability expectations were associated with higher job demands, role conflicts, and work-family conflicts, as well as poorer indicators of health outcomes.

### 4.1 Concerning research question 2

Findings converge on a buffering amplifying pattern along the work-related extended availability work-family conflict health outcome sequence. Organizational resources, such as supportive leadership, family-supportive culture, social belonging, job autonomy, and schedule control, generally mitigate conflict and downstream strain, while individual resources, such as boundary control, attenuate day-level spillover from information and communication demands. Interpreted through the core mechanisms of the JD-R Model (Bakker et al., 2023), work-related extended availability functions as a job demand triggering the health-impairment pathway, while organizational and individual resources activate the motivational pathway by buffering these effects. From a Conservation of Resources perspective (Buchwald and Hobfoll, 2004), these resources help interrupt resource loss cycles that are typically triggered by sustained work-related extended availability demands. As for the mediation pathways, the JD-R Model (Bakker et al., 2023) and Conservation of Resources Theory (Buchwald and Hobfoll, 2004) offer a theoretical framework for contextualizing the examined findings. After analyzing all the studies included in the final sample, a crucial impact of the job and social resources was indicated. Particularly speaking, supervisory behaviors (Stempel et al., 2022) and social resources (Allgood et al., 2022) were indicated as significant moderators. This aligns with JD-R Model’s differentiation between job demands and job resources (Bakker et al., 2023), where the latter can mitigate the strain-inducing mechanisms activated by work-related extended availability. Importantly, from the perspective of the JD-R Model (Bakker et al., 2023), some factors may be interpreted as both a resource and a demand depending on the context. This refers to the use of information and communication technology for work-related communication outside work, while the numerous individual characteristics may influence the impact of the work-related extended availability on work-family conflict. Organizational conditions play a crucial role in mitigating the adverse effects of work-related extended availability on work-family conflict and health outcomes. Haar and Wilkinson (2023) demonstrated that structural autonomy and workplace flexibility are vital in reducing work-family conflict and enhancing health outcomes. Their study indicated that older employees might experience more pronounced negative effects from work-related extended availability, but supportive organizational practices can buffer these effects. Similarly, Allgood et al. (2022) found that social belonging and instrumental leadership are essential in mitigating burnout by alleviating conflicts between work and family-life activities. Based on data collected during the COVID-19 pandemic, this finding, emphasizes the importance of horizontal organizational resources during periods of disruption. From the perspective of the Work-Life Border Theory (Clark, 2000), such organizational conditions help shape the permeability of boundaries between work and nonwork domains, allowing employees to maintain clearer separations that reduce conflict. Schieman and Young (2013) established that the effects of work-related extended availability included the organization of work environment instructions and individual job characteristics. They documented job resource factors (i.e., autonomy and schedule control) that buffered effects of work-related communications from outside regular working hours. Additionally, Gadeyne et al. (2018) pointed out that an individual’s preferences are always contingent on organizational factors, given that several reasons, such as the work context, a high integration norm, or high demands, can override the preferences. This pattern reflects central assumptions of the Conservations of Resources Theory (Buchwald and Hobfoll, 2004) in so far that strong demands and integration norms accelerate resource loss and reduce the compensatory potential of job resources. Thus, while investigating the effects of work-related information and communication technology use on work-family conflict, it is equally important to consider the appropriate work context. According to Martineau and Trottier (2023), autonomy and feedback from the job lead to rigid and impermeable boundaries. Employees could therefore use segmentation-based boundary management strategies that reduced work-family conflict, but heightened availability demands. From the Conservation of Resources Theory perspective, losing resources is particularly obvious to people facing significant job demands. There is a buffering effect of job resources against the harmful career interference caused by using work-related technologies during non-work time. However, this effect is reduced in environments with strong integration preferences or high job demands (Martineau and Trottier, 2023). Martineau and Trottier (2023) also provided evidence of the important role of job design, in terms of autonomy and job feedback interrelating with work-life boundaries and conflict during mandatory telework. They argued that boundary enactment strategies can move toward segmentation, which is less efficacious in minimizing inter-role conflict. In line with Table 2, this evidence prioritizes organizational resources and individual boundary control as key levers to reduce work-family conflict and downstream health outcomes. Overall, these findings support the JD-R model (Bakker et al., 2023) and Conservations of Resources Theory (Buchwald and Hobfoll, 2004) by showing that work-related extended availability operates as a demand depleting personal resources, while organizational and social factors buffer its negative impact. Consistent with Work-Life Border Theory (Clark, 2000), these results further highlight how organizational norms and individual boundary preferences jointly determine the permeability between work and nonwork domains.

### 4.2 Concerning research question 3

Concerning research question 3, the available evidence of studies investigating segmentation preferences in this context is limited (Bauwens et al., 2020; Becker and Lanzl, 2023; Martineau and Trottier, 2023; Thörel et al., 2021).

For RQ3a, the identified studies did not find a consistent moderation of the link between work-related extended availability and health outcomes by segmentation preferences. On the one hand, the results highlight the dual nature of workplace flexibility. While it can intensify work by blurring the boundaries between work and personal life, it can also enhance health outcomes by providing workers with greater autonomy. Hence, various factors affect how work-related extended availability influences work-family conflict and health outcomes. As Thörel et al. (2021) suggest: “It is possible that for some of the employees in our sample work-related extended availability constituted a way of dealing with high work demands” (Thörel et al., 2021, p. 16). Becker and Lanzl (2023) explained that while both segmenters and integrators are exposed to work-induced technological demands, the intrusion that segmenters experience substantially impacts their health outcomes. This finding demonstrates the importance of considering individual differences in boundary management preferences when developing, designing, and implementing digital communication methods and practices. As Adams and Schwarz (2024) explained, while some individuals may prefer to keep work and personal life separate, others are more flexible and allow work and personal realms to blend. Segmenters, as they prefer to keep strong boundaries, are particularly sensitive to the intrusions of digital tools. Integrators, as they adopt a less strict view of work-life boundaries, are less impacted by intrusions, as Becker and Lanzl (2023) suggest. As noted above, these associations were not universally confirmed, as Bauwens et al. (2020) and Thörel et al. (2021) did not find evidence of moderating effects of segmentation preferences on the relationships between work-related extended availability and health outcomes. On the other hand, Bauwens et al. (2020) and Thörel et al. (2021) did not found any evidence that segmentation preferences moderated the relationship between work-family conflict and health outcomes. Insofar, the effect of segmentation preferences on the direct association between work-related extended availability and health outcomes remain open, and future research needs to clarify the buffering or amplifying potential of segmentation preferences in this context.

For RQ3b, none of the reviewed studies (Bauwens et al., 2020; Becker and Lanzl, 2023; Martineau and Trottier, 2023; Thörel et al., 2021) directly tested the proposed moderated mediation via work-family conflict. Notably, the moderated mediation specified in RQ3b therefore remains an untested theoretical assumption that has to be addressed in future research. Complementing the gap, the study by Becker and Lanzl (2023) reported channel-specific differences earlier in the chain, with information and communication technology use relating to technology-related demands by segmentation preference, but this does not constitute a direct test of the full RQ3b pathway.

Taken together, the mixed and largely null evidence for RQ3a and the absence of direct tests for RQ3b indicate a substantive conceptual and empirical gap to be addressed in future work.

### 4.3 Implications for high-reliability organizations

Beyond the general business and organizational context, and although the included studies identified contextual and individual moderators in the links between work-related extended availability, work-family conflict, and health outcomes, empirical work specific to high-reliability organizations remains scarce (e.g., Scholarios et al., 2018; Vila, 2006). Personnel in high-reliability organizations operate under conditions of constant vigilance, high risk, and minimal tolerance for error, with staff often expected to respond immediately to emergencies regardless of official working hours. Scholarios et al. (2018) offered police-specific evidence on work-related extended availability through the lens of shift extensification, that is, unplanned extensions to rostered shifts that reduce employees’ control over working time. Drawing on a 2011 survey of four UK police forces (*n* = 3,257) and qualitative analysis of 2,198 open-text comments, the authors documented frequent deviations from working-time recommendations and reported that such patterns were associated with work-family conflict, perceived stress, and diminished well-being, often driven by employer-led flexibility and stringent availability expectations in policing. Similarly, Vila (2006) documented fatigue-related health impairments and performance risks stemming from prolonged and erratic work hours in U. S. police departments. Vila (2006) synthesized epidemiological, administrative and field date from 379 patrol officers in four medium sized US police departments and concluded that long and irregular work hours, overtime and insufficient sleep are common in policing and are linked to cumulative fatigue, impaired performance and increased negative health outcomes. The findings (Scholarios et al., 2018; Vila, 2006) underscore that in high-reliability organizations, extended availability not only blurs work-life boundaries but also poses tangible safety and health risks, making boundary-aware scheduling and recovery-supportive organizational cultures particularly critical. Table 2 highlights mediators and moderators identified in this systematic review. Work-family conflict, job autonomy, schedule control, and supervisory role modeling are central mediators and moderators between work-related extended availability and health outcomes. These factors appear especially relevant in high-reliability settings. The combination of irregular schedules, high cognitive and emotional demands, and expectations of rapid responsiveness, together with cultural norms emphasizing duty, resilience, and commitment, can make those organizations personnel particularly vulnerable to the negative effects of work-related extended availability. Integrating the insights from Scholarios et al. (2018) and Vila (2006) suggest that loss of schedule control (e.g., shift extensification) and fatigue-related resource depletion may amplify the pathways identified in Table 2, pointing to unique vulnerability patterns in policing and similar high-risk occupations. Studying work-related extended availability in these environments is therefore not only of academic value but also crucial for developing targeted measures to reduce work-family conflict and protect recovery and, in turn, improve health outcomes. In that context, rigid availability norms and high job demands are likely risk factors, whereas family supportive leadership, peer cohesion, schedule control and boundary aware communication are plausible buffers. Although few studies (e.g., Scholarios et al., 2018; Vila, 2006) directly examined work-related extended availability in policing, military, and other high-risk settings, related literatures point to relevant levers. Law enforcement agencies face risks to officer and community safety and organizational reputation; practical risk-management tools are increasingly emphasized, though many agencies remain early in adoption (Hutto, 2009). Parallel work underscores the need for psychological support to address stress and mental ill-health prevalent in high-risk jobs (Stelnicki et al., 2021). Evidence from shift work and risk management suggests plausible mechanisms and intervention targets even in the absence of direct studies on work-related extended availability. Drawing on these mechanisms, future research in high-reliability organizations could examine whether loss of schedule control, for example through shift extensification, amplifies the indirect effect of work-related extended availability on health outcomes via work-family conflict. Furthermore, it seems plausible that recovery-supportive supervisory behaviors buffer the negative impact of work-related extended availability on emotional exhaustion, with this buffering effect being stronger in high-reliability organizations than in general organizational contexts.

## 5 Limitations

While our study provides valuable insights, several limitations should be acknowledged:

- Heterogeneity in Research Designs and Sample Sizes: The studies included in this review employed different research designs and sample sizes, which may contribute to heterogeneity in the findings. This variability can affect the consistency and generalizability of the results. Since we adopted a literature review framework, we did not employ meta-analytical methodologies to assess issues such as heterogeneity or bias statistically. Future research could benefit from a meta-analysis to provide a more robust assessment of these factors (Petticrew and Roberts, 2006).
- Use of Self-Report Scales: Many of the examined studies relied on self-report scales, which are inherently subject to biases such as social desirability and recall bias (Podsakoff et al., 2012). Participants may overestimate or underestimate their experiences, potentially skewing the results. This reliance on self-reported data is a common limitation in psychological and social research and should be considered when interpreting the findings.
- Risk of Bias in Participant Selection: Some studies evaluated the risk of bias in participant selection as moderate due to their sampling strategy and cross-sectional design. Non-probability samples can limit the representativeness of the findings and reduce the power of statistical analyses, making it difficult to generalize the results to broader populations (Boyd et al., 2023).
- Exclusion Criteria and Search Terms: This literature review’s limitations also stem from the exclusion criteria and specific search terms used during the literature search process. Focusing on particular keywords and setting strict inclusion and exclusion criteria may have inadvertently omitted some relevant studies, potentially biased the synthesis of findings, and limited the scope of the review.
- Moderating Impact of Segmentation Preferences: The moderating impact of segmentation preferences on the relationship between work-related extended availability and health outcomes was not universally confirmed across all studies. This inconsistency suggests that the effect of segmentation preferences may be less robust than initially implied and warrants further investigation in future research.

## 6 Implications

Building on our synthesis (Table 2), we propose two priorities for high-reliability settings. First, stricter availability norms and work-related extended availability increase work-family conflict and are associated with poorer health outcomes. These effects are weaker when family supportive leadership and peer cohesion are high. Second, boundary aware communication design attenuates the effect of work-related extended availability on work-family conflict and health outcomes, particularly among employees with higher boundary control.

To address the limitations, future research should consider the following directions:

- Standardization of Measures: Developing and utilizing standardized measures for assessing work-related extended availability and related constructs could enhance the reliability and comparability of findings across studies.
- Larger and More Diverse Samples: Studies with larger and more diverse samples could improve the generalizability of the findings and provide more robust insights into work-related extended availability’s impacts.
- Longitudinal and Experimental Designs: Employing longitudinal and experimental research designs could help elucidate the causal relationships between work-related extended availability, work-family conflict, and health outcomes, offering deeper insights into their temporal dynamics.
- Exploration of Additional Moderators: Investigating other potential moderators, such as organizational culture, social support, and specific job characteristics, could provide a more comprehensive understanding of the factors influencing the relationship between work-related extended availability and health outcomes.

By addressing these limitations and pursuing these research directions, future studies can build on this review’s findings to develop more effective strategies and interventions for mitigating the adverse effects of work-related extended availability and promoting better health outcomes for employees. The theoretical implications of our study are substantial.

This nuanced understanding challenges overly pessimistic views that see work-related extended availability as inherently harmful and overly optimistic perspectives that downplay its potential negative consequences.

From a practical standpoint, our findings suggest several strategies that organizations might adapt to mitigate the potential negative effects of work-related extended availability. First, recognizing and respecting individual differences in boundary management preferences is crucial. Rather than imposing uniform expectations regarding availability, organizations should strive to accommodate diverse approaches to boundary management. Second, enhancing employees’ sense of control over boundaries, for instance, through clear communication about expectations, respect for personal time, and flexible policies, may help reduce the negative impact of work-related extended availability. Third, ensuring employees have access to adequate resources, including supportive supervision and social support networks, can buffer against the potential strain of work-related extended availability.

## 7 Conclusion

In conclusion, our review contributes to a more nuanced understanding of the relationship between work-related extended availability and employee health outcomes by highlighting the complex interplay of individual, organizational, and contextual factors that shape this relationship. Rather than viewing work-related extended availability as inherently beneficial or detrimental, our findings suggest that its impact depends on how it is structured, experienced, and managed. Addressing our research questions, the evidence indicates that higher work-related extended availability is generally associated with increased work-family conflict and, in turn, poorer health outcomes, with work-family conflict emerging as the most proximal and robust mediator. For RQ2, organizational and job resources mitigate these links. Family-supportive supervisory behavior and social belonging reduce conflict and downstream strain, and job autonomy/schedule control buffer effects of work-related extended availability, whereas strong availability expectations and high demands can erode these protections. For RQ3, evidence for segmentation preferences as a moderator is mixed. No moderation could be found on the extended-availability health link and a moderated mediation via work-family conflict has not been tested in the included studies.

By integrating multiple theoretical perspectives, we offer a more comprehensive understanding of the complex relationship between work-related extended availability and employee health outcomes than is possible through any theoretical lens. Our findings suggest that this relationship is neither deterministic nor straightforward, but rather is shaped by a complex interplay of individual preferences, organizational factors, and contextual variables.

## Data Availability

The original contributions presented in the study are included in the article/Supplementary material, further inquiries can be directed to the corresponding author.
