# Supplementary material for: Always available? A systematic review on extended work-related availability, health outcomes and work-family conflict
Source: Front Psychol. 2026 Jan 12;16:1726421. doi: 10.3389/fpsyg.2025.1726421 (PMC12833971; doi:10.3389/fpsyg.2025.1726421)
Supplement: Supplementary file 1 [file Table_1.docx]

**Supplementary Table S1. Overview of included studies (N = 17).**

| Article | Research Subject | Design | Sample Characteristics | Key Variables | Key Findings |
| --- | --- | --- | --- | --- | --- |
| Adams and Schwarz (2024) | Examining the effects of digitally extended availability on work-family conflict in remote work settings, moderated by work demands and gender | Two-wave moderated mediation analysis | Participants were employed parents in heterosexual relationships in Germany, working at least 20 hours per week (N = 1,983) | Digitally extended availability; Work demands; Work-family conflict; Gender differences | Working from home is associated with increased work-family conflict through higher digitally extended availability. Higher work demands further strengthen this mediation effect for fathers, but not for mothers |
| Allgood et al. (2022) | Examining how organizational resources reduce burnout during remote work by mitigating work-family conflict | Cross-sectional survey with mediation analysis | Participants were local government employees from a large U.S. city, 8.24 years of work experience, 50.80% females (N = 124) | Work-related extended availability, Psychological detachment, Sleep, Exhaustion, Segmentation preferences | A negative cross-lagged association between Work-related extended availability and psychological detachment. No indirect effects of work-related extended availability on sleep or exhaustion through detachment were found. There was no evidence of interindividual differences in the effects of work-related extended availability on the outcomes |
| Bauwens et al. (2020) | Examining how technology acceptance and work-related information and communication technology use after hours affect work-life balance among teachers | Cross-sectional survey with structural equation modeling | Participants were secondary school teachers in Flanders (Belgium), 42.22 average age, 64.30% females (N = 288) | Technology acceptance; Work-related information and communication technology use after hours, Work-life balance, Integration preference, Digital learning environments, Social influence, Performance expectancy | Social influence reduces teachers' work-life balance mediated by work-related information and communication technology use after hours. Performance expectancy of digital learning environments and integration preference were associated with higher work-life balance. No support was found for the moderating role of integration preference |
| Bayazit and Bayazit (2019) | Examining how flexible work arrangements affect work-family conflict and health through flexibility individualized arrangements (i-deals) and family-supportive culture | Cross-sectional survey | Participants were white-collar managers and professionals from various industries in Istanbul, 8.90 years of work experience, 31.50 average age, 43.00% females (N = 213) | Flexible work arrangements, flexibility i-deals, family-supportive cultures, work-family conflict, perceived general health | Flexible work arrangements are linked to reduced work-to-family conflict through flexibility i-deals; family-supportive cultures predict conflict in both directions and moderate the effect of i-deals on family-to-work conflict; work-to-family conflict mediates the relationship of flexibility i-deals and family supportive cultures with general health |
| Becker and Lanzl (2023) | Examining technology-driven work-to-family stress and boundary management preferences during the COVID-19 pandemic | Longitudinal study during COVID-19 pandemic | Participants were working adults in Germany, 46.93 average age, 41.13% females (N = 637) | Boundary management, segmentation preference, technostress, work-to-family stress, techno-stressors (interruptions, invasion, overload) | The study found significant differences in how segmenters and integrators experience techno-stressors. Communication technologies cause work-to-family stress, with interruptions, invasion, and overload being critical factors. The results offer theoretical insights into the boundary- transcending effects of technology use during remote work conditions and provide recommendations for employer practices |
| Cho et al. (2020) | Examining how daily information and communication technology demands at work and after work impact work-family conflict, considering negative affect and boundary control | Daily diary study with multilevel mediation analysis | Participants were full-time employees from various industries in the U.S., 35.41 average age (N = 98) | Daily information and communication technology demands, work-family conflict, negative spillover, role conflict, negative affect, boundary control | Different types of information and communication technology demands at work and after work have unique impacts on work-family conflict. Extended after-work availability increases work-family conflict, but this effect is mitigated by high boundary control. Negative affect mediates the effect of on-the-job information and communication technology demands on work-family conflict |
| Choi et al. (2022) | Examining how after-hours work-related communication technology use influences work-family conflict, stratified by gender and working time | Cross-sectional survey analysis | Participants were waged workers in Korea with a spouse and at least one child (N = 17,426) | Work-related communication technology use outside working hours, work interference with family, family interference with work, work-family conflict | The study found that using communication technology after work increases the risk of work-family conflict among South Korean workers with children, especially when working long hours, with women facing a 20% higher risk than men |
| Derks et al. (2016) | Examining how off-job work-related smartphone use affects work-family conflict and family role performance, depending on segmentation preference | Four-day quantitative diary study | Participants were Dutch employees with smartphone access, 37.8 average age, 44% females (N = 71) | Work-related smartphone use during off-job time, work-family conflict, family role performance, segmentation preference | For integrators, more frequent work-related smartphone use during off-job time is associated with better family role performance through reduced work-family conflict. For segmenters, smartphone use has no impact on work-family conflict and family role performance |
| Elshaer et al. (2024) | Examining how off-job work-related internet use affects quality of life through work-family conflict and off-job control | Cross-sectional survey analyzed with PLS-SEM | Participants were faculty members at tourism and hospitality faculties in Egypt, 55.40% females (N = 341) | Work-related mobile internet usage, work-family conflict, off-job control, quality of life | Work-related smartphone use during off-job hours and work-family conflict negatively impact quality of life. Higher off-job control buffers these negative effects and results in better quality of life outcomes |
| Gadeyne et al. (2018) | Examining how work-related information and communication technology use outside work hours affects work-to-home conflict, moderated by integration preference, organizational norms, and work demands | Cross-sectional survey | Participants were Flemish working parents with at least one child under 12 years, 36.84 average age, 85.00% females (N = 467) | Work-related information and communication technology -use (smartphone vs. PC/laptop) outside work hours, integration preference, integration norms, work demands, work-to-home conflict | Work-related PC/laptop use outside work hours is positively related to work-to-home conflict. This effect is buffered for individuals with a high integration preference, but only when organizational integration norms and work demands are low. No effects were found for smartphone use |
| Haar and Wilkinson (2024) | Examining the effects of mobile work during family time on conflict and well-being, moderated by age | Moderated mediation analysis | Participants were employed adults in New Zealand, 34.11 average age, 38.20% females (N = 422) | Smart device use for work, work-family conflict, family-work conflict, well-being outcomes (job anxiety, job depression, insomnia), age as a moderator | Smart device use for work positively influences work-family and family-work conflict and well-being outcomes. Work-family and family-work conflict mediate the relationship between Smart device use for work and well-being outcomes, and age moderates these effects, with older workers experiencing higher conflict |
| Knardahl and Christensen (2021) | Examining home-based work and after-hours availability in relation to well-being and organizational outcomes | Cross-sectional analyses | Participants were 13,119 office employees from Norway, for availability analyses, a subsample of 5,228 was included. | Work factors previously associated with health complaints, mental distress, positive affect, work-private life conflict, commitment, and intention to leave | Working from home was cross-sectionally linked to job demands, role stressors, and work-private life conflict, while availability expectations were additionally associated with health complaints, sleep problems, and lower support. No prospective effects emerged |
| Martineau and Trottier (2022) | Examining how boundary enactment and work design features influence work-life conflict during mandatory telework | Survey-based study during COVID-19 telework period | Participants were remote employees of a Canadian accounting association, working 32-35 hours per week, 82.80% females (N = 93) | Work design features (autonomy, job feedback), work-life boundary enactment (segmentation), work-life conflict | Work design characteristics that foster inflexible boundaries support segmentation boundary enactment, which in turn helps decrease work-life conflict. Although segmentation does not always mediate the relationship, it consistently minimizes inter-role conflict |
| Schieman and Young (2013) | Examining the effects of after-hours work contact on distress, sleep problems, and work-to-family conflict, moderated by job demands and resources | Cross-sectional study using data from the 2011 Canadian Work, Stress, and Health Study | Participants were working adults from a national Canadian labour force survey, 40.00 average age, 48.00% females (N = 5,729) | Work-related communications outside of regular hours (Work Contact), work-to-family conflict, psychological distress, sleep problems, job autonomy, schedule control, challenging work, job pressure | Work Contact was associated with higher levels of work-to-family conflict, psychological distress, and sleep problems. The association was weaker among workers with more job autonomy, schedule control, and challenging work, and stronger among those with more job pressure, with work-to-family conflict contributing to these effects |
| Stempel et al. (2022) | Examining how extended availability affects performance through work-family conflict, moderated by supervisor role modeling | Cross-sectional online survey study | Participants were employed psychology students in Germany, working an average of 41.81 hours per week, 43.00% females (N = 258) | Extended availability demands, supervisory role modeling, emotional exhaustion, work-family conflict, job performance | High work-life-friendly role modeling by supervisors attenuated the detrimental indirect effect of extended availability demands on performance via work-family conflict. Supervisors are identified as key figures for interventions related to extended availability demands |
| Thörel et al. (2021) | Examining the effects of work-related extended availability on detachment, sleep, and exhaustion | Cross-lagged panel design with three waves | Participants were recruited via WiSoPanel, 60.6% females, 47.9 average age, various sectors (N = 528) | Work-related extended availability, psychological detachment, sleep problems, exhaustion, segmentation preferences | A cross-lagged negative association was found between work-related extended availability and detachment. No indirect relationship was found between work-related extended availability and either sleep or exhaustion via detachment. No evidence was found for interindividual differences in the effects of work-related extended availability on any of the outcomes |
| Wright et al. (2014) | Examining how communication technology use outside work hours influences work-life conflict and employee well-being | Online survey of 168 employees from more than 30 companies | Participants were full-time employees from more than 30 companies in a Midwestern U.S. city, 37.64 average age, 66.00% females (N = 168) | Communication technology use outside of regular work hours, work-life conflict, burnout, job satisfaction, turnover intentions, attitudes toward communication technologies | Hours of work-related communication technology use outside regular work hours contributed to work-life conflict, but positive attitudes toward such technologies predicted decreased work-life conflict. Work-life conflict predicted burnout and job satisfaction but not turnover intentions, controlling for age, perceived life stress, and attitudes |
